# Chat-based artificial intelligence for patient information on atrial fibrillation and cardiac implantable electronic devices: comment

**DOI:** 10.1093/europace/euad377

**Published:** 2023-12-29

**Authors:** Hinpetch Daungsupawong, Viroj Wiwanitkit

**Affiliations:** Private Academic Consultant, Lak 52 Phonhong, Vientiane 10000, Lao People's Democratic Republic; Research Center, Chandigarh University, Mohali, India

Dear editor, we hereby discussed on the publication ‘Accuracy and comprehensibility of chat-based artificial intelligence for patient information on atrial fibrillation and cardiac implantable electronic devices.^1^ By asking questions of Google Bard, Bing Chat, and ChatGPT Plus, three distinct natural language processing and chat interfaces, the study assessed their appropriateness, comprehensibility, appearance of confabulation, lack of pertinent content, and recommendations for clinically relevant decisions. Furthermore, the Flesch Reading Ease score and word count were used to gauge how readable the responses were.

Determining the relative strengths and disadvantages of each natural language processing (NLPC) interface is challenging because the study did not compare the interfaces’ performance with a baseline or control group. The total number of questions answered in the interfaces is not stated in the study, therefore, it is not apparent if the sample size is large enough to draw firm conclusions. The results would have been more reliable and more broadly applicable with a bigger sample size. The study provides the proportion of relevant content that is missing for each NLPC interface, but it does not analyse or explain why specific elements were absent. Future advancements might have benefited from knowing the causes of the inadequacies.

The study’s future course may involve correcting these shortcomings by adding a control group, expanding the sample size, and carrying out a more thorough examination of the content that is lacking. To give a more thorough review of the NLPC interfaces’ performance, the study could also investigate additional assessment criteria including user satisfaction and reaction time. However, it should be emphasized that the user of the artificial intelligence (AI) system ultimately choose whether or not to adhere to a just and moral norm.^2^

## References

[1] Hillmann HAK, Angelini E, Karfoul N, Feickert S, Mueller-Leisse J, Duncker D. Accuracy and comprehensibility of chat-based artificial intelligence for patient information on atrial fibrillation and cardiac implantable electronic devices. Europace 2023:euad369. 38127304 10.1093/europace/euad369 PMC10824484

[2] Kleebayoon A, Wiwanitkit V. ChatGPT, critical thing and ethical practice. Clin Chem Lab Med 2023;61:e221. 37247851 10.1515/cclm-2023-0495

